# NF-κB induces miR-148a to sustain TGF-β/Smad signaling activation in glioblastoma

**DOI:** 10.1186/1476-4598-14-2

**Published:** 2015-02-11

**Authors:** Hui Wang, Jian-Qing Pan, Lun Luo, Xin-jie Ning, Zhuo-Peng Ye, Zhe Yu, Wen-Sheng Li

**Affiliations:** Department of Neurosurgery, The Third Affiliated Hospital, Sun Yat-Sen University, 600 Tian He Road, Tian He District, Guangzhou, Guangdong 510630 China; Department of Neurosurgery, The Affiliated Shenzhen Nanshan Hospital, Guangdong Medical College, Shenzhen, 518052 China; Guangzhou Biocare Cancer Institute, Guangzhou, 510663 China

**Keywords:** miR-148a, QKI, NF-κB, TGF-β, Aggressiveness, Glioblastomas

## Abstract

**Background:**

Inflammatory cytokines and transforming growth factor-β (TGF-β) are mutually inhibitory. However, hyperactivation of nuclear factor-κB (NF-κB) and TGF-β signaling both emerge in glioblastoma. Here, we report microRNA-148a (miR-148a) overexpression in glioblastoma and that miR-148a directly suppressed Quaking (QKI), a negative regulator of TGF-β signaling.

**Methods:**

We determined NF-κB and TGF-β/Smad signaling activity using pNF-κB-luc, pSMAD-luc, and control plasmids. The association between an RNA-induced silencing complex and *QKI*, mitogen-inducible gene 6 (*MIG6*), S-phase kinase–associated protein 1 (*SKP1*), and glyceraldehyde-3-phosphate dehydrogenase (*GAPDH*) mRNA was tested with microribonucleoprotein immunoprecipitation and real-time PCR. Xenograft tumors were established in the brains of nude mice.

**Results:**

QKI suppression induced an aggressive phenotype of glioblastoma cells both *in vitro* and *in vivo*. Interestingly, we found that NF-κB induced miR-148a expression, leading to enhanced-strength and prolonged-duration TGF-β/Smad signaling. Notably, these findings were consistent with the significant correlation between miR-148a levels with NF-κB hyperactivation and activated TGF-β/Smad signaling in a cohort of human glioblastoma specimens.

**Conclusions:**

These findings uncover a plausible mechanism for NF-κB–sustained TGF-β/Smad activation via miR-148a in glioblastoma, and may suggest a new target for clinical intervention in human cancer.

**Electronic supplementary material:**

The online version of this article (doi:10.1186/1476-4598-14-2) contains supplementary material, which is available to authorized users.

## Introduction

Glioblastoma multiforme (GBM) is the most common and lethal primary brain tumor in adults; it has a spectrum of aberrantly aggressive phenotypes [1]. Although non-metastasizing, and the despite advances in treatments over the past decades, the extensive invasion of GBM limits patient survival to approximately 12–14 months [2, 3]. The extremely poor prognosis of patients with GBM is due to the ability of GBM to diffusely infiltrate the cerebral cortex, which limits the extent of surgical resection and high-dose radiotherapy for fear of unacceptable permanent neurological damage to the patient. Poor blood–brain barrier penetration, intrinsic GBM resistance, and nonselective toxicity restrict the value of traditional chemotherapy [4]. Therefore, the development of improved therapies rests on further understanding of the molecular mechanism of the aggressive malignant phenotype of GBM. However, this remains largely unclear.

Transforming growth factor-β (TGF-β) is a multifunctional polypeptide that can switch from being a tumor suppressor in normal or dysplastic cells to a tumor promoter in advanced cancers [5–7]. Although TGF-β is a notable tumor suppressor in most cases, it promotes proliferation, invasion, metastasis, and intratumoral angiogenesis in non-epithelial cancer such as glioma [8–15]. Interestingly, TGF-β usually suppresses nuclear factor-κB (NF-κB) activity in normal cells, and NF-κB activation induces Smad7 expression, which in turn inhibits TGF-β signaling through Smads [16, 17]. However, NF-κB and TGF-β pathway activation both emerge in glioma [18]. This indicates the possibility of cross-talk between NF-κB and TGF-β signaling in cancer, which remains poorly understood.

The RNA-binding protein Quaking (QKI) belongs to the signaling transduction and activation of RNA (STAR) protein family [19]. The three QKI isoforms, QKI-5, QKI-6, and QKI-7, which share an RNA-binding hnRNPK homology (KH) domain, can dimerize with one another and shuttle between the cytoplasm and the nucleus [20, 21]. The *QKI* gene is implicated as being important in schizophrenia, and QKI controls the translation of many oligodendrocyte-related genes [22, 23]. QKI expression is also a characteristic of glial progenitors, and a high frequency of deletion of chromosome 6q26-27, containing QKI, was observed in anaplastic astrocytoma and GBM [24–27]. Chen and team validated the finding that QKI suppresses GBM by stabilizing microRNA-20a (miR-20a), which targets TGF-β receptor 2 (TGFβR2) [28]. The potential importance of QKI in GBM pathogenesis is elevated further by its direct regulation by the tumor suppressor TP53 [29]. However, the frequency of TP53 mutations is about 70% in GBM [30], which means that TP53 may not regulate QKI in most cases. Taken together, we suspect that there may be an alternative regulatory mechanism of QKI protein expression in GBM.

Being able to coordinately regulate target gene repertoires, miRNAs can potentially modulate multiple steps of cancer development and progression [31, 32]. From analysis using a published microarray-based high-throughput assessment, we found that miR-148a expression is significantly higher in GBM tissues than in normal brain tissue. Herein, we report that miR-148a was induced by NF-κB and directly targeted and suppressed the 3′ untranslated regions (3′ UTRs) of multiple genes that function as negative regulators of TGF-β, leading to TGF-β hyperactivation and GBM aggressiveness. These results identified a regulatory mechanism that results in sustained TGF-β activation in human GBM, thereby supporting the functional and clinical significance of epigenetic events in cancer progression.

## Results

### Reduced QKI levels in glioblastoma correlated with patient prognoses

**Figure 1:**
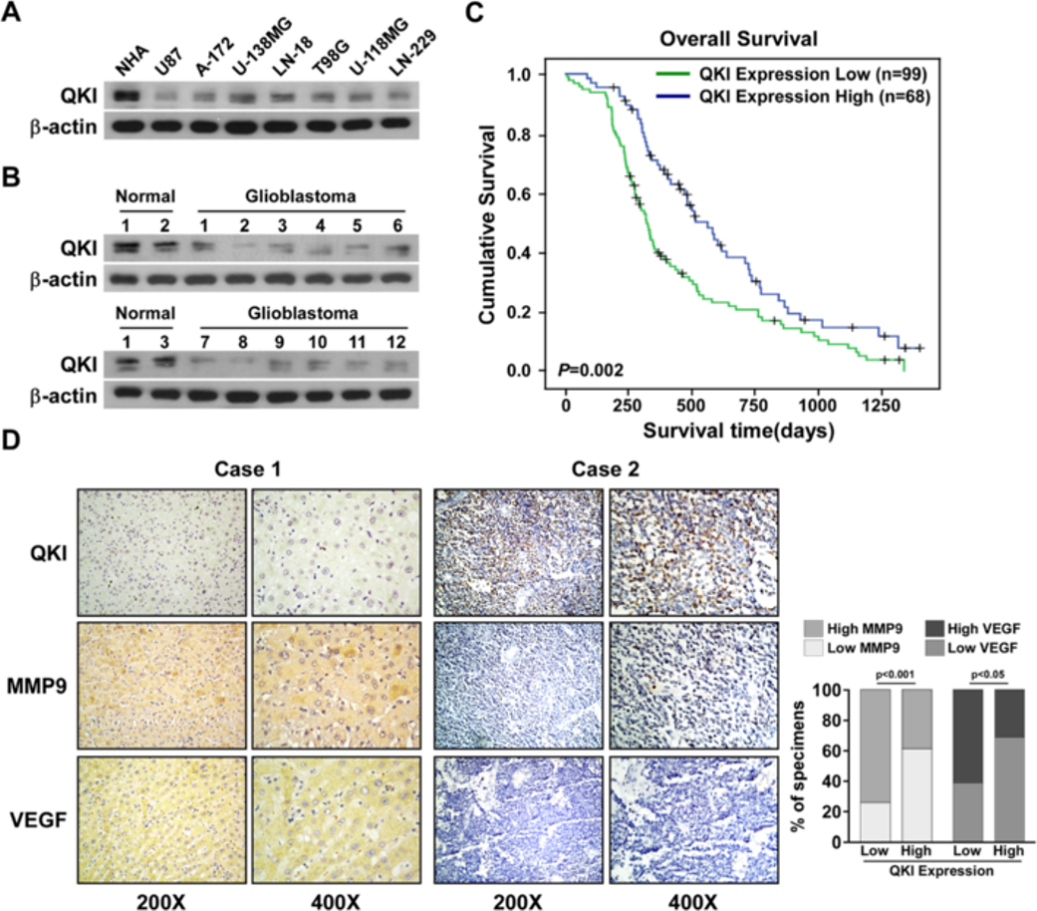
**Restoration of QKI inhibits glioma tumorigenesis.** Western blotting (WB) of QKI in **(A)** three normal brain tissues and 12 glioma tissues, and **(B)** NHAs and seven glioblastoma cell lines. β-Actin was used as the loading control. **(C)** Kaplan–Meier analysis of QKI expression in survival of patients with glioblastoma (*P* = 0.002, log-rank test; *n* = 167). **(D)** QKI expression was inversely associated with VEGF and MMP9 expression in 167 clinical glioma specimens. Figures are visualizations of two representative cases and percentages of samples with low or high QKI expression relative to VEGF or MMP9 levels.

QKI reduction is associated with the initiation and progression of glioblastoma [28, 33], but its clinical significance in glioblastoma remains unexplored. Immunoblotting analysis showed that QKI expression was reduced in all seven glioblastoma cell lines and in glioblastoma tissues (*n* = 12) compared with that in primary NHAs and normal brain tissues (*n* = 3) (Figure 1A and B). Furthermore, statistical analysis revealed that QKI levels were associated with shorter overall survival in patients with glioblastoma (*P* = 0.002) (*n* = 167; Figure 1C). Additionally, we found that QKI expression inversely correlated with MMP9 (*P* < 0.001) and VEGF levels (*P* < 0.05) (Figure 1D). These data suggest a possible link between QKI reduction and human glioblastoma progression.

### MiR-148a targeted QKI

*QKI* is located on chromosome 6q26-27, which is frequently deleted in astrocytoma and glioblastoma. Interestingly, integrative analysis using the cBioPortal for Cancer Genomics (http://cbioportal.org) indicated that approximately 70% of the *QKI* gene is not deleted in glioblastoma (Additional file 1: Figure S1A). Given the *QKI* promoter hypomethylation (Additional file 1: Figure S1B), DNA methylation is unlikely to be the major mechanism responsible for the downregulation of QKI. Chen *et al* found that TP53 regulates QKI directly; however, most TP53 mutations in glioblastoma do not feature QKI deletion (Additional file 1: Figure S1C). Moreover, real-time PCR analysis revealed no appreciable alteration of *QKI* mRNA expression in glioblastoma tissue compared with normal brain tissue (Additional file 1: Figure S1D), which suggests that the reduction of QKI protein in glioblastoma is not due to transcriptional inhibition.

**Figure 2:**
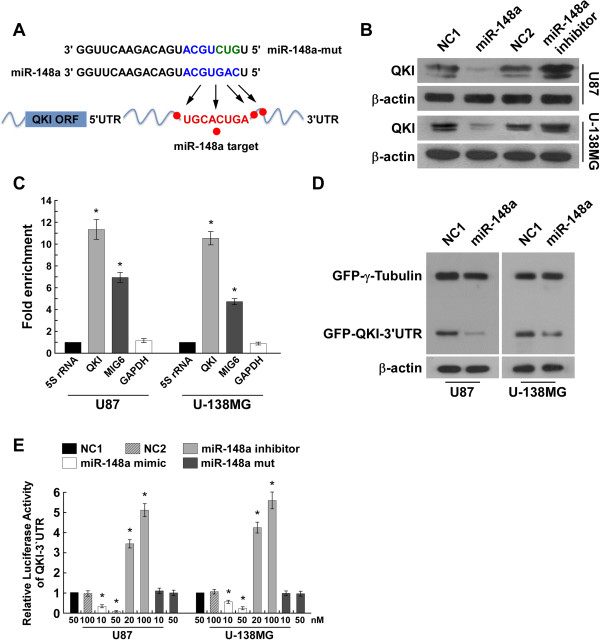
**miR-148a directly targets QKI. (A)** Predicted miR-148a target sequence in QKI-3′UTR and mutant containing three mutated nucleotides in the seed sequence of miR-148a (miR-148a-mut). **(B)** WB of QKI expression in negative control 1 (NC1)- or miR-148a–transduced cells or cells transfected with NC2 or miR-148a inhibitor. β-Actin was used as the loading control. **(C)** MiRNP IP revealing the association of miR-148a with QKI. MIG6 and GAPDH were used as positive and negative controls, respectively; 5S rRNA was used as a control for overall expression levels. **(D)** WB analysis of GFP expression. **(E)** Luciferase assay of cells transfected with pGL3-QKI-3′UTR reporter with miR-148a mimic (10 or 50 nM), miR-148a mutant, or miR-148a inhibitor (20 or 100 nM).

Analysis using three publicly available algorithms (TargetScan, PicTar, miRanda) revealed that the QKI-3′UTR contains conserved critical nucleotides that may serve as a legitimate target of miR-148a (Figure 2A), which is substantially overexpressed in glioblastoma [34]. Immunoblotting analysis showed that QKI expression was decreased in miR-148a–transduced cells and increased in miR-148a inhibitor–transfected cells compared to the negative control cells (Figure 2B). The microribonucleoprotein (miRNP) IP assay revealed a selective association of miR-148a with *QKI* (Figure 2C). Ectopically expressing miR-148a had no effect on GFP–γ-tubulin expression, but robustly inhibited the expression of GFP with a complete, wild-type QKI-3′UTR in glioblastoma cells (Figure 2D). Notably, the luciferase reporter assay showed that miR-148a inhibitor abolished the inhibitory effect of miR-148a linked with QKI-3′UTR, whereas miR-148a into which a mutation had been introduced lost the ability to reduce luciferase activity despite the presence of QKI-3′UTR (Figure 2E). Collectively, these results demonstrate that QKI is a *bona fide* target of miR-148a.

### MiR-148a targeted SKP1 and activated TGF-β signaling

**Figure 3:**
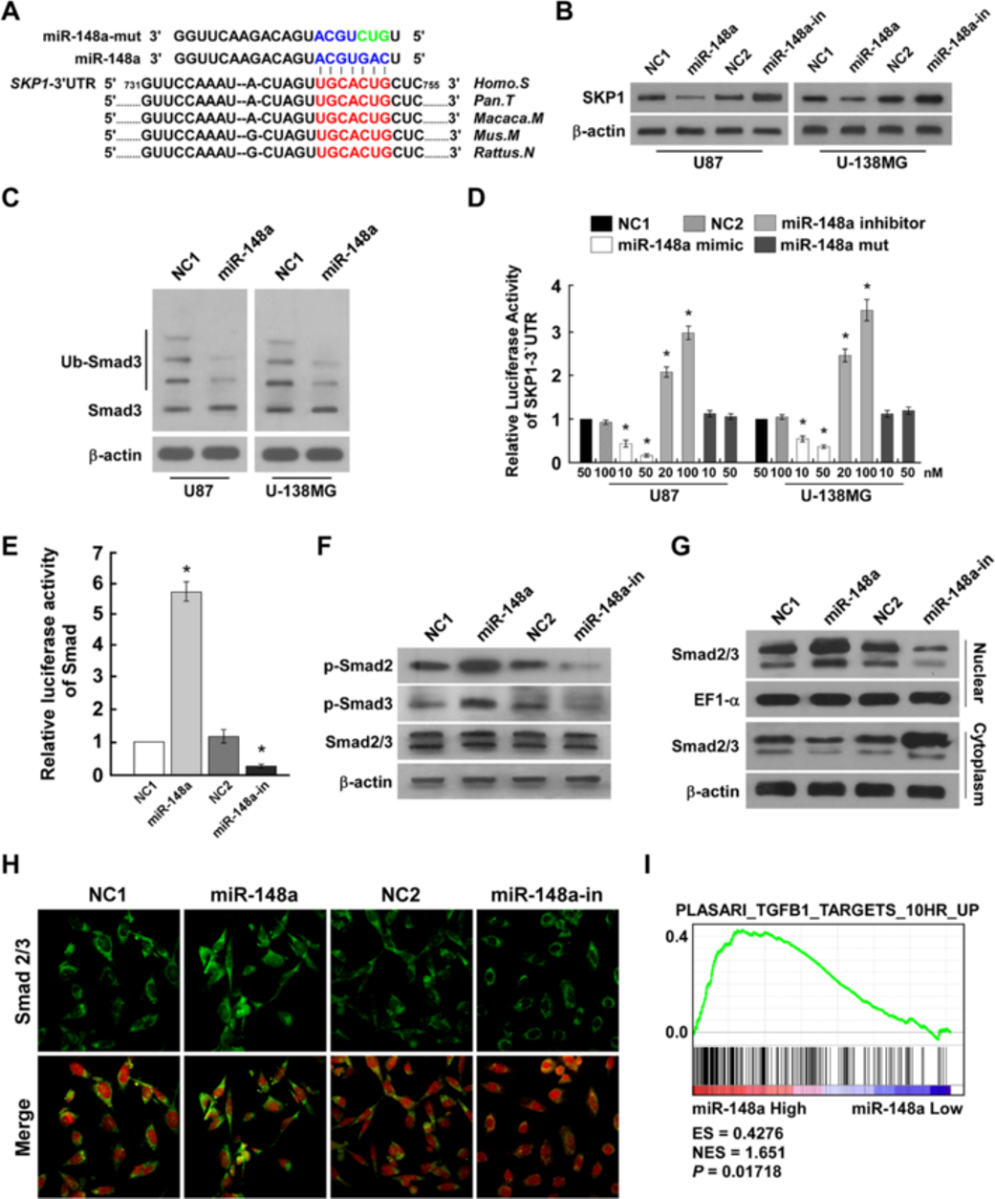
**MiR-148a directly targets SKP1 and activates TGF-β signaling. (A)** Predicted miR-148a target sequence in SKP1-3′UTR and mutant containing three mutated nucleotides in the seed sequence of miR-148a (miR-148a-mut). **(B)** WB of SKP1 expression in negative control 1 (NC1)- or miR-148a–transduced cells or cells transfected with NC2 or miR-148a inhibitor. β-Actin was used as the loading control. **(C)** WB analysis of ubiquitinated Smad3 (Ub-Smad3) expression. **(D)** Luciferase assay of cells transfected with pGL3-SKP1-3′UTR reporter with miR-148a mimic (10 or 50 nM), miR-148a mutant, or miR-148a inhibitor (20 or 100 nM). **(E)** Smad luciferase reporter activity. **(F)** WB analysis of p-Smad2, p-Smad3, and Smad/3 expression. **(G)** WB analysis of Smad2/3 expression in different cellular localizations. **(H)** IF analysis of Smad2/3 expression. **(I)** GSEA plot showing that miR-148a expression correlated positively with TGF-β–activated gene signatures (PLASARI_TGFB1_TARGETS_10HR_UP) in published TCGA patient gene expression profiles (*n* = 538).

As QKI has been reported as a negative regulator of TGF-β signaling, we investigated whether miR-148a is involved in TGF-β activation. TGFβR activation results in the phosphorylation of specific receptor-activated Smads (R-Smads), which translocate into the nucleus and regulate the transcription of target genes [35–37]. Smad3 is one of the key R-Smads that is degraded by an E3 ubiquitin ligase complex, ROC1-SCF^Fbw1a^, which consists of ROC1, SKP1, cullin 1 (CUL1), and F-box protein (Fbw1a) [38]. Interestingly, *SKP1* is the theoretical target gene of miR-148a (Figure 3A). As predicted, ectopic expression of miR-148a in U87 and U138MG cells decreased SKP1 expression and ROC1-SCF^Fbw1a^ ubiquitination activity (Figure 3B and C). Furthermore, miR-148a overexpression reduced the luciferase activity of SKP1-3′UTR in a consistent and dose-dependent manner, but miR-148a inhibition increased it. However, transfection of the miR-148a-mut, containing mutations in the miR-148a seed region, did not decrease the luciferase activity of SKP1-3′UTR (Figure 3D). Taken together, our results demonstrate that SKP1 is also a *bona fide* target of miR-148a in glioblastoma. Furthermore, miR-148a overexpression increased Smad luciferase reporter activity of Smad2/3 phosphorylation, while miR-148a inhibition reduced it (Figure 3E and F). Immunoblotting analysis and immunofluorescence (IF) staining revealed that miR-148a–expressing cells exhibited a marked increase in nuclear Smads, which was confirmed by the cellular fraction assays (Figure 3G and H). Lastly, but no less importantly, analyzing miR-148a expression and TGF-β–regulated gene signatures via GSEA of published TCGA patient expression profiles allowed us to confirm that miR-148a expression levels were positively correlated with the TGF-β–activated gene signatures (Figure 3I).

### MiR-148a overexpression correlated with glioblastoma progression

**Figure 4:**
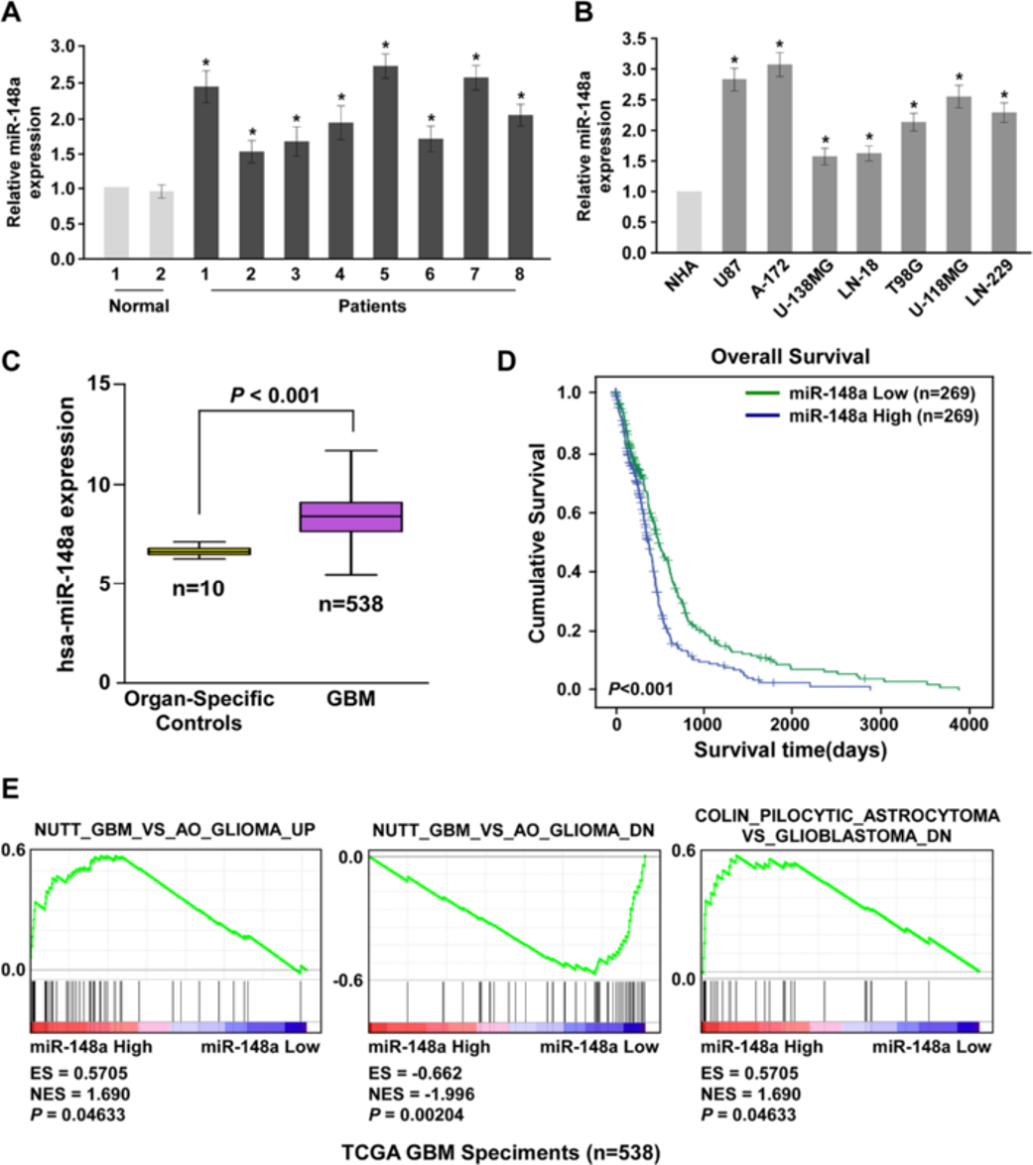
**MiR-148a is overexpressed in gliomas.** Real-time PCR analysis of miR-148a expression in **(A)** three normal brain tissues and 12 glioma tissues, and **(B)** NHAs and seven glioblastoma cell lines. Transcript levels were normalized to U6 expression. **(C)** MiR-148a upregulation was identified in 548 patients from the microarray data using published TCGA data. **(D)** Correlation between miR-148a levels and survival by Kaplan–Meier analysis of patients with low (<median, *n* = 269) or high (>median, *n* = 269) miR-148a expression. **(E)** GSEA plot showing that miR-148a expression correlated positively with glioma progression gene signatures in published TCGA patient gene expression profiles (*n* = 538). Each bar represents the mean ± SD of three independent experiments. *P < 0.05.

Real-time PCR analysis revealed that miR-148a was markedly overexpressed in the eight primary glioblastoma tissues compared to that of the normal brain tissues, and in the seven glioblastoma cell lines compared with the NHAs (Figure 4A and B). Consistent with this, microarray analysis showed that miR-148a was significantly upregulated in glioblastoma (Figure 4C). Furthermore, statistical analysis revealed that miR-148a levels were inversely correlated with survival (*P* < 0.001, Figure 4D). Importantly, miR-148a expression also correlated with the expression of glioblastoma progression–related gene signatures (Figure 4E), suggesting that miR-148a plays significant roles in glioblastoma progression and that its levels are associated with poor overall survival in patients with glioblastoma.

### MiR-148a upregulation augmented glioblastoma aggressiveness *in vitro*and *in vivo*

**Figure 5:**
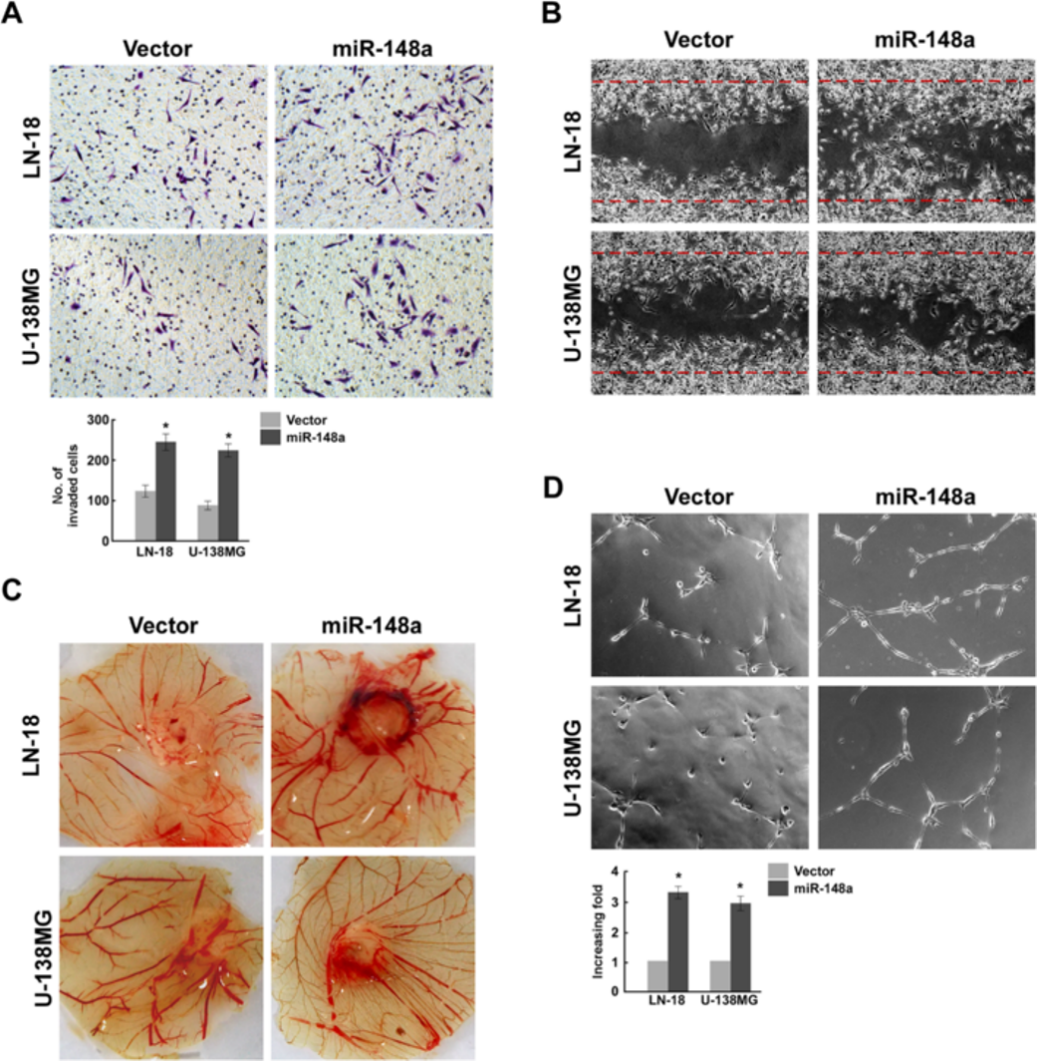
**MiR-148a promotes the aggressive phenotype of glioblastoma cells** ***in vitro*** **. (A)** Representative images and quantification of invading cells analyzed by Transwell matrix penetration assay. **(B)** Representative images of vector- or miR-148a–transfected glioblastoma cell lines analyzed by wound healing assay. **(C)** Representative images of chicken CAM blood vessels stimulated with conditioned medium. **(D)** Representative images and quantification of HUVECs cultured on Matrigel-coated plates with conditioned medium from vector- or miR-148a–transfected glioblastoma cell lines.

As glioblastomas are highly angiogenic and generally kill via invasiveness, we investigated whether miR-148a could modulate glioblastoma cell angiogenesis and invasiveness. As shown in Figure 5A and B, miR-148a overexpression dramatically increased both LN18 and U138MG cell migration and invasiveness. Meanwhile, miR-148a overexpression strongly promoted the ability of glioblastoma cells to induce vessel formation (CAM, Figure 5C and D). Moreover, suppressing miR-148a yielded results to the contrary (Additional file 2: Figure S2A and S2D). Thus, these results support the view that miR-148a overexpression is linked to glioblastoma progression.

**Figure 6:**
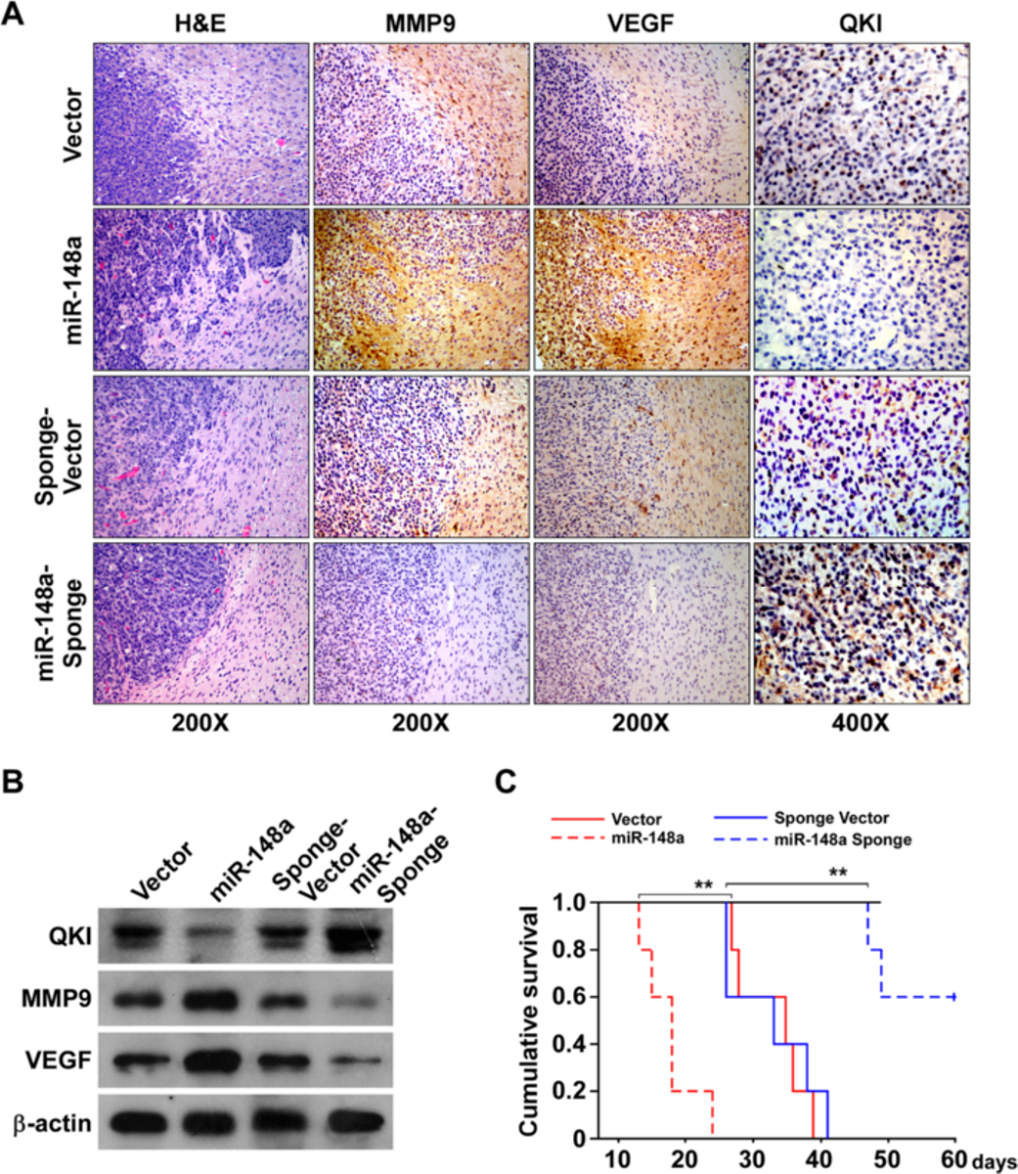
**miR-148a promotes the aggressive phenotype of glioblastoma cells** ***in vivo*** **. (A)** H&E and IHC staining showed that miR-148a overexpression induced the aggressive phenotype of glioblastoma cells *in vivo*, while miR-148a suppression inhibited it, as indicated by MMP9- and VEGF-positive cells and glioblastoma cell invasion into the surrounding brain tissues. Figures are representative images of various brain sections from two independent experiments with five mice per group with similar results. **(B)** WB analysis of MMP9, VEGF, and QKI expression in brain tissues. **(C)** Kaplan–Meier survival of mice (*n* = 5 per group) inoculated with the indicated cells. Error bars represent the mean ± SD of three independent experiments. *P < 0.05.

The biological role of miR-148a in promoting the aggressive phenotype of glioblastoma was further examined *in vivo* by stereotactically implanting engineered glioblastoma cells into the brains of nude mice. We used a stable miRNA sponge to inhibit miR-148a *in vivo*. Notably, the borders of miR-148a–overexpressing tumors exhibited spike-like structures invading into the surrounding brain tissues, whereas the control tumors exhibited sharp edges (Figure 6A), indicating that miR-148a overexpression induced glioblastoma cell invasion into the brain. Meanwhile, IHC and immunoblotting revealed that MMP9 and VEGF expression was upregulated in miR-148a–overexpressing tumors, while that of QKI was downregulated; the effects were attenuated in miR-148a–inhibited tumors (Figure 6A and B). More importantly, Kaplan–Meier analysis demonstrated that mice bearing miR-148a–overexpressing glioblastomas had significantly shorter survival than control animals; in contrast, mice bearing miR-148a–inhibited tumors survived longer than the control mice (Figure 6C). Taken together, our results suggest that miR-148a induced glioblastoma cell invasiveness and angiogenesis *in vitro* and *in vivo*.

### QKI and SKP1 played important roles in miR-148a–induced glioblastoma cell invasiveness and angiogenesis

**Figure 7:**
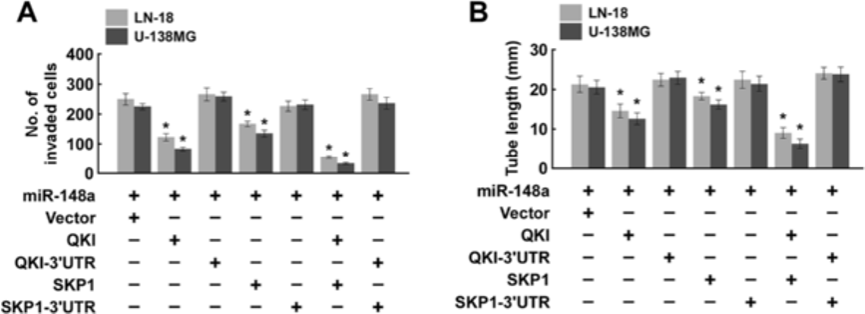
**QKI and SKP1 play important roles in miR-148a promotion of the aggressive phenotypes of glioblastoma. (A)** Invaded glioblastoma cells transfected with different nucleotides. **(B)** The tube length of glioblastoma cells transfected with different nucleotides.

In the attempt to understand the role of QKI and SKP1 repression in miR-148a–induced invasiveness and angiogenesis, the effects of QKI and SKP1 (without and with the 3′ UTRs) were examined in miR-148a–overexpressing cells. Co-transfecting QKI or SKP1 with miR-148a significantly reduced glioblastoma cell invasiveness and decreased the ability of glioblastoma cells to induce HUVEC tube formation (Figure 7A and B). However, combining QKI-3′UTR or SKP1-3′UTR with miR-148a in the glioblastoma cells had no obvious effect compared with transfecting glioblastoma cells with only miR-148a. Taken together, our results suggest that suppressing QKI and SKP1 plays an important role in miR-148a promotion of invasiveness and angiogenesis.

### NF-κB induced miR-148a in glioblastoma

**Figure 8:**
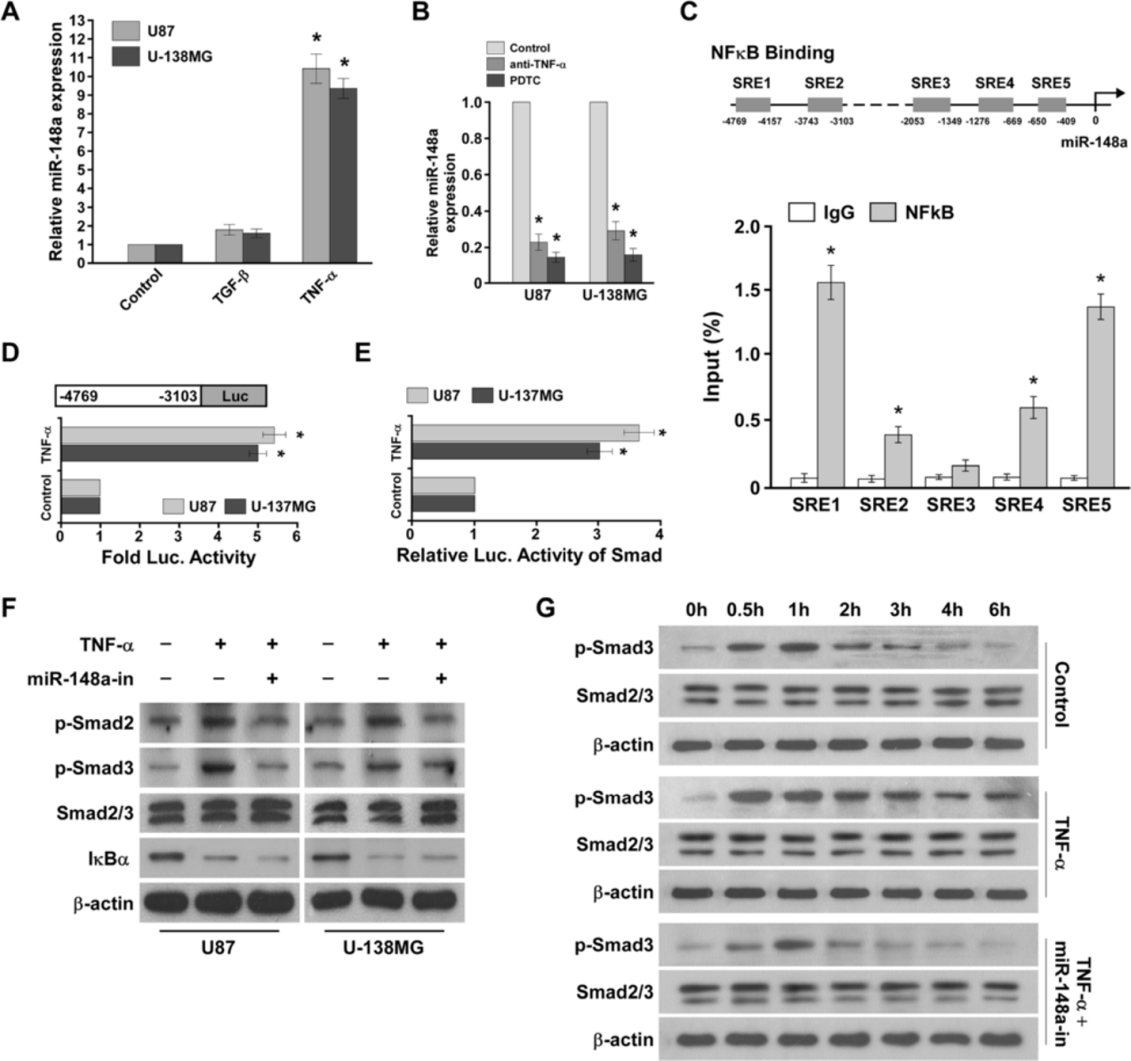
**NF-κB induces miR-148a expression.** **(A)** Real-time PCR analysis of miR-148a expression in glioblastoma cells treated with 10 ng/mL TNF-α or 100 pM TGF-β for 3 hours. Transcript levels were normalized to U6 expression. **(B)** Real-time PCR analysis of miR-148a expression in cells treated with or without PDTC or neutralizing anti–NF-κB antibody (2 μg/mL) for 3 hours. **(C)** Schematic of typical miR-148a promoter SREs. Also shown are ChIP assay results for the miR-148a promoter SREs physically associated with NF-κB. ChIP assay results for the SREs of *MIR148a* promoter physically associated with NFκB or RNA pol II in indicated cells treated with or without TNF-α (see Supplemental Figure 3). **(D)** Luciferase assay of cells transfected with the pGL3-SRE reporter treated with or without TNF-α. **(E)** Smad luciferase reporter activity in cells treated with or without TNF-α for 3 hours **(F)** WB of p-Smad2, p-Smad3, Smad2/3, and IκBα in TNF-α–treated cells (10 ng/mL) in response to treatment with control. **(G)** *In vitro* kinase assay indicating that TGF-β–induced endogenous Smad activity was prolonged in TNF-α–treated cells, which the miR-148a inhibitor abrogated. Error bars represent the mean ± SD of three independent experiments. *P < 0.05.

It is notable that miR-148a expression also correlated with the expression of NF-κB–regulated gene signatures (Additional file 3: Figure S3A). However, ectopically expressing miR-148a had no effect on the NF-κB luciferase reporter activity (Additional file 3: Figure S3B). Interestingly, there was a marked increased in miR-148a expression in glioblastoma cells treated with tumor necrosis factor-α (TNF-α), whereas TGF-β had minimal effects on miR-148a expression (Figure 8A). Importantly, pyrrolidine dithiocarbamate (PDTC, a NF-κB inhibitor) and a TNF-α–neutralizing antibody prevented the stimulatory effect of TNF-α on miR-148a (Figure 8B). Analysis of the *MIR148A* promoter region using ConSite (http://consite.genereg.net/) predicted five typical NF-κB–responsive elements (serum response elements, SREs; Figure 8C). Chromatin immunoprecipitation (ChIP) showed that endogenous NF-κB proteins significantly bound to the first SRE in the *MIR148A* promoter (Figure 8C). We also subcloned the fragment containing the two NF-κB binding sites into the pGL3 dual luciferase reporter vectors. As shown in Figure 8D, TNF-α increased the luciferase activity. Taken together, our result indicates that the NF-κB pathway induced miR-148a expression by directly targeting the *MIR148A* promoter. Furthermore, Smad luciferase reporter activity was significantly increased in TNF-α–treated glioblastoma cells, as expected (Figure 8E). As shown in Figure 8F, p-Smad2/3 were also elevated in TNF-α–treated cells, but miR-148a inhibitor halted the effect. Moreover, TGF-β–induced endogenous Smad2/3 activity was prolonged in TNF-α–treated glioblastoma cells (Figure 8G), which suggests that TNF-α sustained TGF-β–induced TGF-β/Smad activation in glioblastoma cells. These results indicate that miR-148a was involved in TNF-α–mediated TGF-β/Smad activation.

### MiR-148a expression correlated with NF-κB activity and TGF-β/Smad pathway hyperactivation in clinical glioblastoma

**Figure 9:**
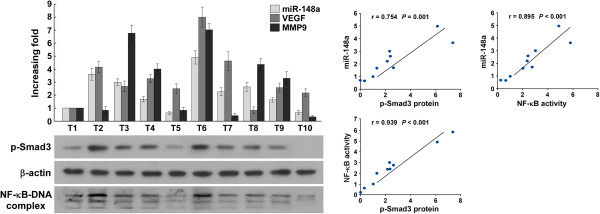
**Clinical relevance of the NF-κB/miR-148a/TGF-β/Smad axis in human glioblastoma.** Expression and correlation of miR-148a with *MMP9* and *VEGF* mRNA expression, p-Smad3 expression, and NF-κB activity (detected by an EMSA assay) in 10 human glioblastoma samples.

Lastly, we examined whether activation of the NF-κB/miR-148a/TGF-β/Smad axis identified in our glioblastoma cell models would also be evident in clinical glioblastoma tumors. As shown in Figure 9, there was positive correlation between the miR-148a levels in 10 freshly collected glioblastoma samples with *MMP9* (*r* = 0.674, *P* < 0.001) and *VEGF* mRNA levels (*r* = 0.748, *P* < 0.001), the DNA-binding activity of NF-κB (*r* = 0.895, *P* < 0.001), and p-Smad3 expression (*r* = 0.754, *P* = 0.001). These results support the notion that hyperactive NF-κB signaling induces miR-148a expression, resulting in TGF-β/Smad pathway activation and consequently leading to the promotion of malignant phenotypes of glioblastoma and poor clinical prognosis of clinical glioblastoma.

## Discussion

The key finding of the present study is that NF-κB increases miR-148a expression to sustain the TGF-β/Smad signaling pathway by downregulating QKI and SKP1. We demonstrate that NF-κB binds directly to the miR-148a promoter to regulate miR-148a expression. Furthermore, QKI and SKP1 are *bona fide* targets of miR-148a. QKI and SKP1 inhibition led to the induction of TGF-β/Smad signaling activity. Ectopic miR-148a expression dramatically promoted glioblastoma aggressiveness both *in vitro* and *in vivo*. Importantly, the significant correlation detected among miR-148a levels, NF-κB, and TGF-β/Smad signaling hyperactivation was confirmed in a cohort of human glioblastoma samples. Hence, the NF-κB/miR-148a/TGF-β pathway represents a critical mechanism for promoting glioblastoma aggressiveness.

QKI is a tumor suppressor in several cancers, including oral cancer, prostate cancer, colorectal cancer, gastric cancer, and brain cancer [28, 39–42]. However, the biological effect of QKI on cancer development and progression remains unclear. In the present study, statistical analysis of clinical specimens revealed, for the first time, that QKI is associated with shorter overall survival of patients with glioblastoma, supporting the notion that QKI functions as a tumor suppressor. In addition to the genome mutations or deletions that can lead to QKI downregulation [27], the loss of QKI expression can also be regulated at the transcriptional level. For example, TP53 regulated QKI expression in glioma cells by directly targeting its promoter [29]. However, the present study found no appreciable alteration of *QKI* mRNA expression in glioblastoma tissues compared with normal brain tissues, which intimates that translational repression might regulate the loss of QKI expression in glioblastoma. Analyses using publicly available algorithms and the present results identified QKI as a direct target of miR-148a in glioblastoma.

Interestingly, we found that NF-κB activation increased the expression of miR-148a, whose promoter contains NF-κB target elements. From the above results, we conclude that NF-κB signaling is probably hyperactivated in glioblastoma, thereby inducing miR-148a expression but reducing QKI expression. In fact, hyperactive signaling correlates with glioma progression and poor prognosis of patients with malignant glioma [43–46]. Thus, the present study uncovers a novel mechanism that regulates QKI expression in glioblastoma.

Despite therapeutic advancements, treating malignant glioma remains a challenge due to ineffective targeting of infiltrating glioma cells and the formation of abnormal, dysfunctional tumor vasculature [11, 47, 48]. TGF-β signaling is highly active in glioblastoma, and elevated TGF-β activity has been associated with poor clinical outcome in this disease [49]. The TGF-β/Smad pathway is considered a therapeutic target in glioma [50, 51]. QKI suppresses TGF-β signaling in glioblastoma, and SKP1 is an essential component of the E3 ubiquitin ligase complex ROC1-SCF^Fbw1a^, which induces Smad3 ubiquitination. As both QKI and SKP1 are *bona fide* targets of miR-148a, we suspect that miR-148a is involved in TGF-β activation. We found that miR-148a enhances the strength and prolongs the duration of TGF-β/Smad signaling. Via TCGA database analysis, we found that miR-148a was significantly associated with shorter overall survival in patients with glioblastoma, and correlated positively with TGF-β/Smad signaling activity (*P* < 0.05). These findings suggest that miR-148a expression could be sufficient for activating TGF-β/Smad signaling.

TGF-β and inflammatory cytokines such as TNF-α and IL-1β are mutually inhibitory. Curiously, the NF-κB and TGF-β signaling pathways are both hyperactive in glioblastoma. Focusing on this issue, Song *et al*. reported that TGF-β induced miR-182 to sustain NF-κB activation in glioma subsets [18]. However, they did not clarify the sustained activation of TGF-β. Sustained activation of regulatory programs requires orchestrated transcription and post-transcriptional regulation of gene expression. Due to their multi-targeting properties and the network effect, miRNAs are perfectly suited to the task. Here, we showed that miR-148a directly repressed QKI and SKP1, which inhibited the TGF-β pathway. Interestingly, we also found that NF-κB induced miR-148a expression. Thus, it is plausible that miR-148a modulates NF-κB–mediated TGF-β activation through multiple mechanisms. On the other hand, analysis of the TCGA datasets indicated that miR-148a promotes glioblastoma aggressiveness. These observations indicate that further investigation of the effect of miR-148a on the TGF-β pathway in glioblastoma is warranted.

## Conclusion

In summary, the present study provides an important link between NF-κB and TGF-β signaling via miR-148a in glioblastoma. Our findings suggest an essential role of miR-148a in regulating GBM cell progression. Understanding the precise role played by miR-148a in GBM progression will not only increase our knowledge of the pathogenesis of gliomas, but also will enable the development of novel therapeutic strategies and the identification of an effective biomarker for predicting outcomes for patients with malignant gliomas.

## Materials and methods

### Cell lines

Primary normal human astrocytes (NHAs) were purchased from ScienCell Research Laboratories (Carlsbad, CA, USA) and cultured according to the manufacturer’s instructions. Glioma cell lines A172, T98G, LN18, LN229, U138MG, U87, and U118MG were from ATCC (Manassas, VA, USA). The cells were grown in Dulbecco’s modified Eagle’s medium supplemented with 10% fetal bovine serum.

### Plasmids, virus production, and target cell infection

The human *MIR148A* gene was PCR-amplified from genomic DNA and cloned into a pMSCV-puro retroviral vector. MiR-148a sponge was constructed by annealing, purifying, and cloning oligonucleotides containing six tandem “bulged” miR-148a–binding motifs into the pMSCV vector. Negative control 1(NC1) is a chemically synthesized double-stranded small RNA used as negative control for miR-148a mimic. NC2 is a chemically synthesized single-stranded RNA molecule used as negative control for miR-148a inhibitor. Both of these negative control molecules exhibit minimum homology to any human, mouse or rat miRNAs annotated in the current released of the miRBase database, and no significant homology to the genomes of those three species as established by sequence alignment. Human *QKI* and *SKP1* (S-phase kinase–associated protein 1) were PCR-amplified from NHA complementary DNA (cDNA) and cloned into the pMSCV vector. The 3′ UTRs of the human *QKI* (QKI-3′UTR) and *SKP1* (SKP1-3′UTR) genes, generated by PCR amplification from NHAs, were cloned into the *Sac*I/*Xma*I sites of pGL3 luciferase reporter plasmid (Promega, Madison, WI, USA) and pEGFP-C3 vector (Clontech, Mountain View, CA USA). The pNF-κB-luc, pSMAD-luc, and control plasmids (Clontech) were used to determine NF-κB and TGF-β/Smad signaling activity. Plasmid transfection was performed using Lipofectamine 2000 (Invitrogen, Carlsbad, CA, USA) according to the manufacturer’s instructions. Stable cell lines expressing miR-148a and miR-148a sponge were generated via retroviral infection using HEK293T cells as described by Li *et al*. [52] and selected with 0.5 μg/mL puromycin for 10 days.

### Tissue specimens and patient information

A total 167 paraffin-embedded, archived clinical glioma specimens comprising World Health Organization (WHO) grade I–IV tumors and 12 freshly snap-frozen glioma tissues were histopathologically diagnosed at the Third Affiliated Hospital of Sun Yat-sen University from 2000 to 2010. Normal brain tissues were obtained from individuals who had died in traffic accidents and who were confirmed to be free of any preexisting pathologically detectable conditions. Prior donor consent and approval from the Institutional Research Ethics Committee were obtained.

### Western blotting analysis

Cells and tissues were harvested in sampling buffer [62.5 mmol/L Tris-HCl (pH 6.8), 10% glycerol, 2% sodium dodecyl sulfate (SDS)] and heated for 5 minutes at 100°C. Protein concentration was determined with the Bradford assay using a commercial kit purchased from Bio-Rad Laboratories (Hercules, CA, USA). Equal quantities of protein were separated electrophoretically on 10% SDS/polyacrylamide gels and transferred onto polyvinylidene difluoride membranes (Roche, Basel, Switzerland). The membranes were probed with diluted antibody. The expression of target proteins was determined with horseradish peroxidase–conjugated anti-rabbit immunoglobulin G (IgG)/anti-mouse IgG (Sigma-Aldrich, St Louis, MO, USA) and enhanced chemiluminescence (Pierce, Rockford, IL, USA) according to the manufacturers’ suggested protocols. The membranes were stripped and reprobed with an anti–β-actin mouse monoclonal antibody (Sigma-Aldrich) as a loading control. The related antibodies were anti-Smad2/3, anti–NF-κB inhibitor (IκBα), anti–matrix metalloproteinase 9 (MMP9), anti–phosphorylated (p)-Smad2, anti–p-Smad3, anti-Smad2, anti–vascular endothelial growth factor (VEGF) (Cell Signaling Technology, Beverly, MA, USA), anti-QKI, and anti–green fluorescent protein (GFP) (Abcam, Cambridge, MA, USA).

### Immunohistochemistry

Immunohistochemical (IHC) analysis was performed to study altered protein expression in 167 clinical glioma tissue sections. In brief, paraffin-embedded specimens were cut into 4-μm sections and baked at 65°C for 30 minutes. The sections were deparaffinized with xylenes and rehydrated. Sections were submerged in EDTA antigenic retrieval buffer and microwaved for antigen retrieval. The sections were treated with 3% hydrogen peroxide in methanol to quench the endogenous peroxidase activity, followed by incubation with 1% bovine serum albumin to block nonspecific binding. The sections were incubated with antibody overnight at 4°C. After washing, the tissue sections were treated with biotinylated anti-rabbit/mouse secondary antibody (Zymed, San Francisco, CA, USA), followed by incubation with streptavidin–horseradish peroxidase complex (Zymed). The tissue sections were immersed in 3-amino-9-ethyl carbazole and counterstained with 10% Mayer’s hematoxylin, dehydrated, and mounted in Crystal Mount (Biomeda, Foster City, CA, USA).

### RNA extraction and real-time quantitative PCR

Total miRNA from cultured cells and fresh surgical glioblastoma tissues was extracted using a mirVana miRNA Isolation Kit (Ambion, Foster City, CA, USA) according to the manufacturer’s instructions. We synthesized cDNA using a TaqMan MicroRNA Reverse Transcription Kit (Applied Biosystems, Foster City, CA, USA) and quantified miR-148a expression using a miRNA-specific TaqMan MiRNA Assay Kit (Applied Biosystems). Real-time PCR was performed using the Applied Biosystems 7500 Sequence Detection System. MiRNA expression was defined based on the comparative threshold (Ct); relative expression levels were calculated as 2 - (Ct miR-148a - Ct U6) after normalization with reference to the quantification of U6 small nuclear RNA expression.

### Microribonucleoprotein immunoprecipitation assay

Cells were cotransfected with a plasmid that encodes hemagglutinin-tagged (HA)-Ago1 and miR-148a (100 nM), followed by HA-Ago1 immunoprecipitation (IP) using an anti-HA antibody. Real-time PCR analysis of the IP material was used to test the association of *QKI*, mitogen-inducible gene 6 (*MIG6*), *SKP1*, and glyceraldehyde-3-phosphate dehydrogenase (*GAPDH*) mRNA with the RNA-induced silencing complex.

### Luciferase assay

Cells (3 × 10^3^) were seeded in triplicate in 48-well plates and allowed to settle for 24 hours. Luciferase reporter plasmids (100 ng) or 100 ng control luciferase plasmid plus 1 ng pRL-TK *Renilla* plasmid (Promega) were transfected into glioblastoma cells using Lipofectamine 2000 (Invitrogen). Luciferase and *Renilla* signals were determined 24 hours after transfection using a Dual Luciferase Reporter Assay Kit (Promega).

### Cell invasion assay

Glioblastoma cells (2 × 10^4^) were plated on the top side of a polycarbonate Transwell filter (with Matrigel) in the top chamber of a BioCoat Invasion Chamber (BD Biosciences, Bedford, MA, USA) and incubated at 37°C for 22 hours; cells in the top chamber were removed with cotton swabs. Cells that had migrated and invaded to the lower membrane surface were fixed in 1% paraformaldehyde, stained with hematoxylin, and counted under a microscope (10 random fields per well, ×100 magnification). The cell counts were expressed as the mean number of cells per field.

### Human umbilical vein endothelial cell tubule formation assay

Matrigel (200 μL; BD Biosciences) was pipetted into each well of a 24-well plate and polymerized for 30 minutes at 37°C. Human umbilical vein endothelial cells (HUVECs) (2 × 10^4^) in 200 μL conditioned medium were added to each well and incubated at 37°C in 5% CO_2_ for 20 hours. Photographs were captured under a × 100 bright-field microscope, and the capillary tubes were quantified by measuring the total lengths of the completed tubule structure. Each condition was assessed at least in triplicate.

### Chorioallantoic membrane assay

Chorioallantoic membrane (CAM) assay was performed using fertilized, day 6 chicken eggs (Yueqin Breeding Co. Ltd., Guangdong, China). A 1-cm wide window was opened on the egg shell and the surface of the dermic sheet on the floor of the air sac was removed to expose the CAM. A 0.5-cm wide filter paper was first placed on top of the CAM, and 100 μL conditioned medium was added to the center of the paper. After closing the window with sterile adhesive tape, the eggs were incubated at 37°C in 80–90% relative humidity for four days. Following fixation with stationary solution (methanol:acetone = 1:1) for 15 minutes, the CAMs were cut and harvested and gross photos of each CAM were taken with a digital camera. The effect of conditioned media harvested from different cultured cells was evaluated based on the number of second- and third-order vessels.

### Intracranial brain tumor xenografts, IHC, and hematoxylin–eosin staining

Glioblastoma cells (5 × 10^5^) were stereotactically implanted into the brains of nude mice (*n* = 5 per group). The mice were monitored daily and euthanized when moribund. Whole brains were removed, paraffin-embedded, sectioned into 4-μm thick slides, and stained with hematoxylin–eosin (H&E) or with anti-QKI (Abcam), anti-MMP9 (Cell Signaling Technology), or anti-VEGF (Cell Signaling Technology) antibodies. Images were captured using an AxioVision Rel. 4.6 computerized image analysis system (Zeiss, Jena, Germany).

### Chromatin immunoprecipitation

Cells (2 × 10^6^) in a 100-mm culture dish were treated with 1% formaldehyde to cross-link proteins to DNA. The cell lysates were sonicated to shear DNA to 300–1000-bp fragments. Equal aliquots of chromatin supernatant were separated and incubated with 1 μg anti–NF-κB (Cell Signaling Technology) or anti-IgG antibody (negative control; Millipore, Billerica, MA, USA) overnight at 4°C with rotation. After reverse cross-linking of protein/DNA complexes to free the DNA, PCR was performed using specific primers.

### Electrophoresis mobility shift assay

Electrophoresis mobility shift assay (EMSA) was performed using a LightShift Chemiluminescent EMSA Kit (Pierce). The following DNA probes containing specific binding sites were used: NF-κB sense, 5′-AGTTGAGGGGACTTTCCCAGGC-3′; NF-κB anti-sense, 5′-GCCTGGGAAAGTCCCCTCAAC-3′.

### Microarray data processing and visualization

Microarray data were downloaded from The Cancer Genome Atlas (TCGA) database (http://cancergenome.nih.gov/). Gene Set Enrichment Analysis (GSEA) was performed using GSEA 2.0.9 (http://www.broadinstitute.org/gsea/).

### Statistics

All statistical analyses were carried out using the SPSS 16.0 statistical software package (SPSS Inc., Chicago, IL, USA). The χ^2^ test was used to analyze the relationship between QKI expression and clinicopathological characteristics. Bivariate correlations between study variables were calculated by Spearman’s rank correlation coefficients. *P* < 0.05 was considered statistically significant.

### Study approval

The Ethical Committee of the Third Affiliated Hospital of Sun Yat-sen University evaluated and approved the use of human glioma tissue specimens, and written informed consent was obtained from all participants or their appropriate representatives. All animal studies were conducted with the approval of the Sun Yat-sen University Institutional Animal Care and Use Committee and were performed in accordance with established guidelines.

## Electronic supplementary material

Additional file 1: Figure S1: Reduction of QKI protein in glioblastoma is not due to transcriptional inhibition. (**A**) cBioPortal analysis results showing that approximately 70% of the *QKI* gene is not deleted in glioblastoma. (**B**) cBioPortal analysis results showing that DNA methylation is unlikely to be the major mechanism responsible for the downregulation of QKI. (**C**) cBioPortal analysis results showing that most TP53 mutations in glioblastoma do not feature QKI deletion. (**D**) Real-time PCR of QKI in 3 normal brain tissues and 12 glioma tissues. (TIFF 548 KB)

Additional file 2: Figure S2: Inhibit miR-148a suppresses aggressive phenotype of glioblastoma cells in vitro. (**A**) Representative images and quantification of invaded cells analyzed using a Transwell matrix penetration assay. (**B**) Representative images of NC-2– or miR-148a inhibtor–transfected glioblastoma cell lines analyzed in a wound healing assay. (**C**) Representative images of chicken chorioallantoic membrane blood vessels stimulated with conditioned medium. (**D**) Representative images and quantification of HUVECs cultured on Matrigel-coated plates with conditioned medium from NC-2– or miR-148a inhibitor–transfected glioblastoma cell lines. (TIFF 4 MB)

Additional file 3: Figure S3: miR-148a expression correlated with the expression of NF-κB–regulated gene. (**A**) GSEA plot showing that miR-148a expression positively correlated with NF-κB–regulated gene signatures in published TGCA patient gene expression profiles (*n* = 538). (**B**) Luciferase-reporter NF-κB activity in the indicated cells. (**C**, **D**) ChIP assay results for the SREs of *MIR148a* promoter physically associated with NFκB or RNA pol II in indicated cells treated with or without TNF-α. Error bars represent mean ± SD from 3 independent experiments. *P < 0.05. (TIFF 600 KB)
