# Supplementary figures and images for: NF-κB induces miR-148a to sustain TGF-β/Smad signaling activation in glioblastoma
Source: Mol Cancer. 2015 Feb 11;14:2. doi: 10.1186/1476-4598-14-2 (PMC4429406; doi:10.1186/1476-4598-14-2)

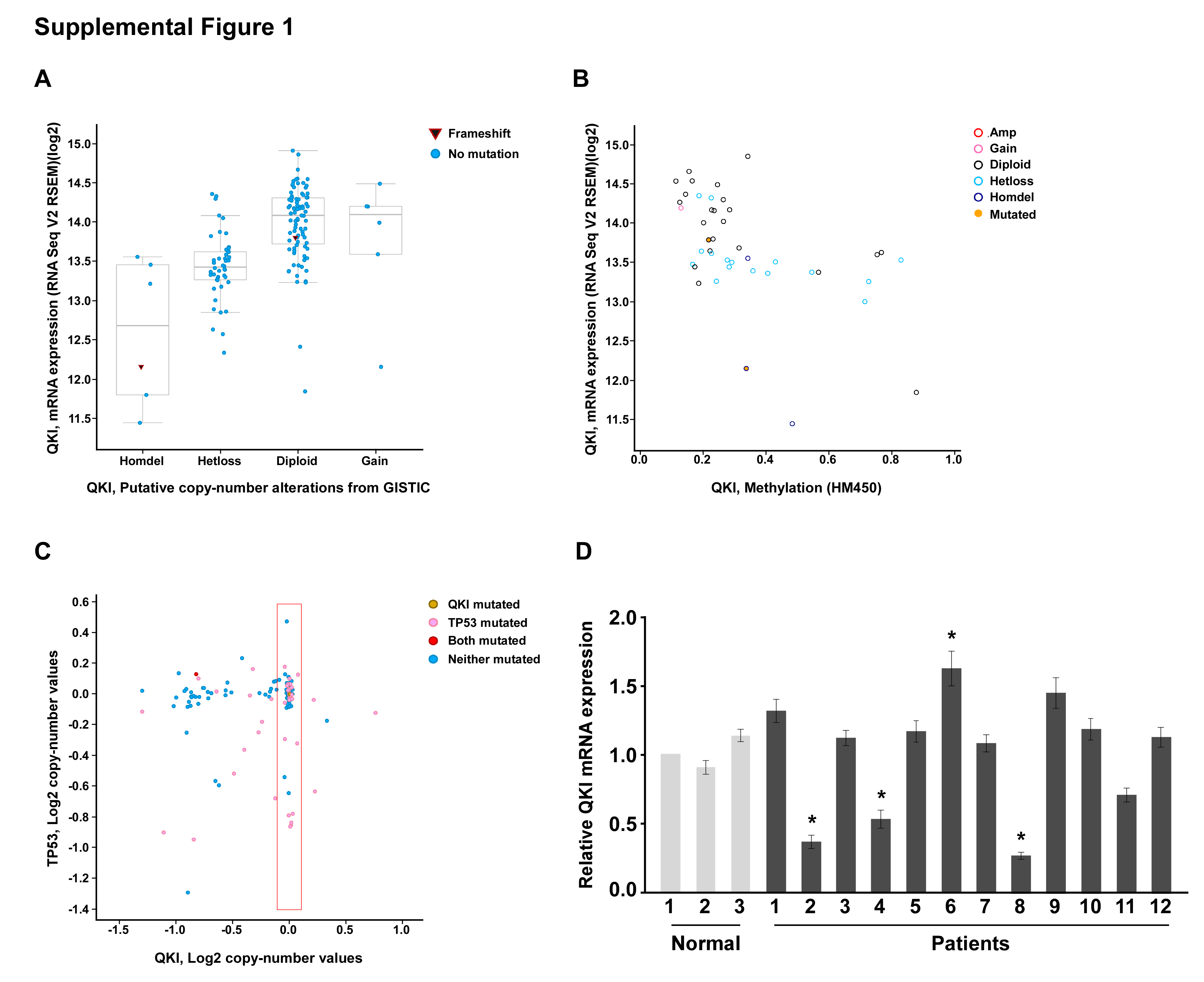

Supplement: Supplementary file 1 — Additional file 1: Figure S1: Reduction of QKI protein in glioblastoma is not due to transcriptional inhibition. (A) cBioPortal analysis results showing that approximately 70% of the QKI gene is not deleted in glioblastoma. (B) cBioPortal analysis results showing that DNA methylation is unlikely to be the major mechanism responsible for the downregulation of QKI. (C) cBioPortal analysis results showing that most TP53 mutations in glioblastoma do not feature QKI deletion. (D) Real-time PCR of QKI in 3 normal brain tissues and 12 glioma tissues. (TIFF 548 KB) [file 12943_2014_1499_MOESM1_ESM.tiff]

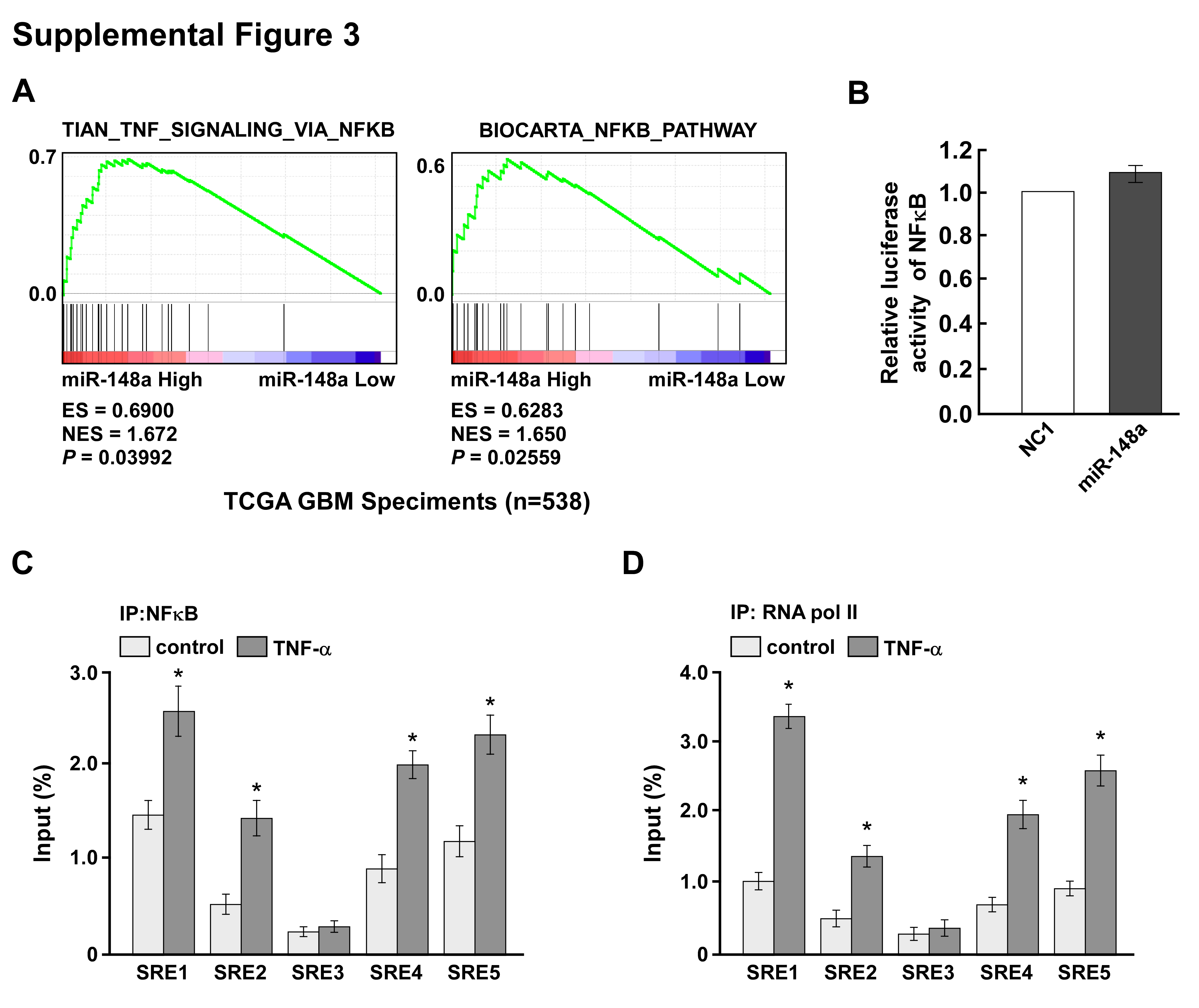

Supplement: Supplementary file 3 — Additional file 3: Figure S3: miR-148a expression correlated with the expression of NF-κB–regulated gene. (A) GSEA plot showing that miR-148a expression positively correlated with NF-κB–regulated gene signatures in published TGCA patient gene expression profiles (n = 538). (B) Luciferase-reporter NF-κB activity in the indicated cells. (C, D) ChIP assay results for the SREs of MIR148a promoter physically associated with NFκB or RNA pol II in indicated cells treated with or without TNF-α. Error bars represent mean ± SD from 3 independent experiments. *P < 0.05. (TIFF 600 KB) [file 12943_2014_1499_MOESM3_ESM.tiff]
